# Distance of the fracture from the radiocarpal surface in childhood: does it determine surgical technique? A retrospective clinical study: A STROBE compliant observational study

**DOI:** 10.1097/MD.0000000000017763

**Published:** 2020-02-14

**Authors:** Gergo Jozsa, Gertrud Devecseri, Peter Vajda, Zsolt Juhasz, Marcell Varga, Tamas Juhasz

**Affiliations:** a Surgical Division of the Department of Paediatrics, Medical School, University of Pécs; b Medical School, University of Pécs; c Sándor Péterfy Street Hospital and Casualty Centre; d Department of Anatomy, Medical School, University of Debrecen, Hungary.

**Keywords:** dia-metaphyseal fracture, ESIN, K-wire, pediatric trauma, short double elastic nailing

## Abstract

Unstable distal metaphyseal and dia-metaphyseal fractures of the radius may have treated with a variety of operative techniques, Kirschner wires (K-wires), dorsally inserted titanium elastic stable intramedullary nailing (DESIN), and short titanium elastic stable intramedullary nailing (SESIN) in children.

The aim of this study was to evaluate the differences in clinical and radiographic outcomes between these methods.

Between January 2009 and December 2017 196 children were treated for forearm fractures in the distal third of the distal radius. Gender of the patients, different types of surgical techniques, number of postoperative X-rays, date of metal removal and degree of axis deviation after the metal removal were studied. Distance of the fracture line from the radiocarpal surface, the width of the distal epiphysis of the radius, and the cumulative width of the distal epiphysis of the ulna and radius were analyzed.

Out of the 196 children, stabilization of the fracture was achieved by K-wire in 139, by DESIN in 44, and by SESIN in 13 patients. The average time of metal removal was significantly shorter (3.8 months), following stabilization with K-wire. In children treated with K-wire, axial deviation of <5° was seen in 118 patients, 5° to 10° deviation in 15 patients, while deviation was above 10° in 6 children. In the DESIN group, <5° axial deviation was found in 37 patients and 5° to 10° in seven patients. In all 13 children treated with SESIN, axial deviation was measured to be <5°. The fracture distance from the radiocarpal surface was on average 23.7 and 45.6 mm in the children treated with K-wire and DESIN, respectively.

Fracture distance from the radiocarpal surface might determine the type of surgical technique required. If the distance of the fracture line is less than the width of the distal radius, osteosynthesis with a K-wire is recommended, while if the distance of the fracture is more than the cumulative width of the radius and the ulna, then DESIN may provide better results. The use of SESIN may be indicated when the area of the growth plate is injured.

## 1 Introduction

Displaced fractures of the distal forearm are the most common injuries in childhood.^[1]^ Traditionally, these fractures have been treated by closed reduction and casting, but, due to the intrinsic instability of these fractures in the cast, many authors prefer surgical treatment to prevent redisplacement.^[2,3]^ Unstable distal forearm fractures are usually treated by closed reduction and minimal invasive fixation. Operative osteosynthesis technique of pediatric wrist fractures is optimally minimally invasive, spare the physis and maintain an acceptable and painless reduction. Unstable, angulated fractures of the distal radius can be treated by several methods: with insertion of Kirschner wires (K-wires),^[4–6]^ with dorsally inserted titanium elastic stable intramedullary nails (DESIN),^[5,7–9]^ or with short titanium elastic stable intramedullary nailing (SESIN).^[10]^ Preliminary result was described by Sinikumpu et al about absorbable elastic nailing of the forearm fractures. This procedure combined with long-arm casting is feasible in treating the pediatric forearm fractures. The technique may bring benefits to handling these challenging fractures. The disadvantages of metallic implants may be avoided. In addition, removal of the implant will not be required. Different types of operative fixation have been advocated, including plates^[11–14]^ and external fixator.^[15]^ Fractures in the area of the dia-metaphyseal junction present a unique problem; they are usually too distal to be treated by classic elastic stable intramedullary nailing and too proximal for conventional K-wire fixation. The optimal treatment for the dia-metaphyseal transition zone is still a matter of debate.

The surgical gold standard for the treatment of distal metaphyseal or dia-metaphyseal fractures of the radius in childhood is closed reduction and K-wire fixation. This method offers several advantages: technically minimally demanding, minimally invasive and does not require any special equipment. Although there are many variations of percutaneous pinning Muller and Kapandji techniques are most commonly used worldwide.^[16]^

Trans-epiphyseal intramedullary K-wire fixation is a treatment option, but this technique does not respect the physis.^[1]^ K-wire fixation also carries many potential complications and disadvantages. K-wire related complications are well known in the literature: migration of the pins, superficial infections, damage of the physeal plates, skin irritation, and insufficient biomechanical stability to maintain the reduction without casting.^[17–20]^ This is the main drawback of this technique: in addition to the discomfort associated with an operation, it is usually also necessary to wear a long or short cast at least for 4 to 6 weeks postoperatively.

Internal fixation with elastic stable intramedullary nailing (ESIN) has increased in popularity.^[21]^ It can improve the quality of a closed reduction but it also can increase the stability of the fixation, thereby avoiding the necessity of cast immobilization and a lower risk of malunion. Disadvantages of ESIN include the potential for injury to structures at the nail entry points, infection (superficial and deep), and the need for subsequent surgery for implant removal. Anterograde ESIN of the radius is not recommended, because this procedure carries a high risk of radial nerve injury. Traditional technique of ESIN differs from the DESIN method. In case of ESIN treatment the insertion point is distal radial metaphysis, as opposed to the DESIN where the nail is inserted from the distal dorsal metaphysis.

SESIN is a modified elastic nailing method for the operative treatment of severely displaced pediatric distal metaphyseal or dia-metaphyseal radial fractures. It is characterized by retrograde insertion of two short pre-bent, elastic titanium nails proximal to the distal radial physis. It provides very stable stabilization without the need for casting. The nails do not touch the physeal plates so the risk of postoperative physeal arrest is reduced.^[10]^ Incorrect insertion points or malpositions of the entry holes may jeopardize the physeal plates, the sensory branch of the radial nerve, or the extensor pollicis longus tendon, which are also complications of the classic ESIN method.^[22–24]^

No data was found in the literature regarding measurement of the distal radius and distal cumulative radius and ulna diameter. There is an absence of information regarding the correlation of the fracture distance and these diameters in the literature.

## 2 Patients and methods

In this study we compared three different surgical techniques for surgical stabilization of unstable fractures of the distal forearm in children and evaluated the differences in clinical and radiographic outcomes between these methods. We retrospectively studied the possible association between the fracture distance from the articular surface and the choice of surgical method.

Clinical application of these techniques have been accepted and permitted in 2009 by our Medical Review Board of the Hungarian Society of Paediatric Surgery and by the Hungarian Paediatric Trauma Committee. Possible benefits and complications were explained to the parents of each child before the procedures. Patients and their caregivers declared their informed consent for publication of study results. Ethical approval was not necessary because of the study form, this investigation was retrospective and based on the measurement on X-rays.

**Figure 1:**
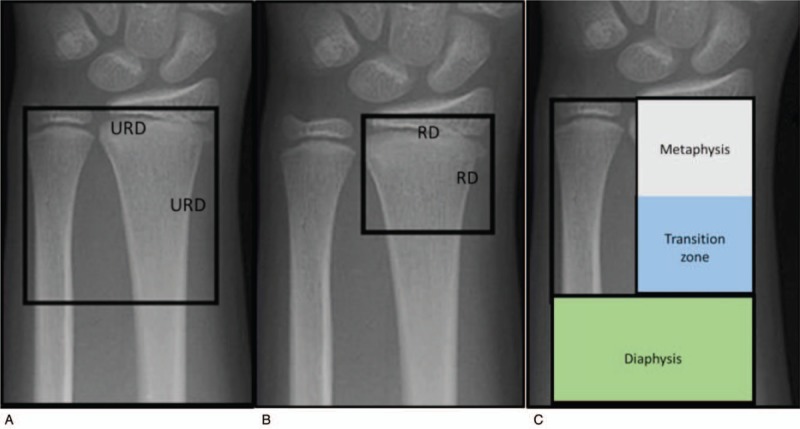
Definition of the large square (a) was based on the cumulative width of the distal epiphysis of the radius and URD, and small square (b) was based on the RD. Definition of the transition zone (c). RD = radius width, URD = ulna radius width.

The dia-metaphyseal transition zone was defined as the large square based on the cumulative diameter of the distal epiphysis of the radius and ulna, minus the small square based on the diameter of the distal epiphysis of the radius alone (Fig. 1.) Between January 2009 and December 2017 a retrospective clinical study was performed at the Surgical Division of the Department of Paediatrics, Medical School, University of Pécs, Hungary. One hundred and ninety-six children were treated for fracture of the forearm in the distal third or isolated fracture of the distal radius. Surgical intervention was indicated if the radius was completely displaced, or the radius showed a 20° angulation or greater and closed reduction alone could not provide stable retention. All fractures were treated by closed reduction and the fixation was performed K-wires, DESIN, or SESIN technique. Inclusion criteria were closed fractures of the distal part of the radius with total radial or dorsal displacement and shortening. We excluded open fractures, physeal injuries, and pathological fractures, and all patients who suffered a fracture in middle or proximal third were excluded.

**Figure 2:**
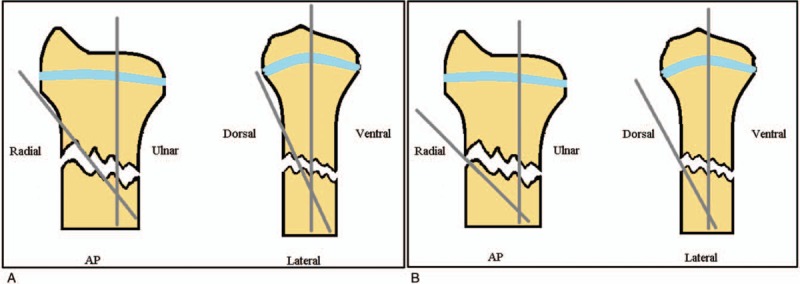
Sematic picture (antero-posterior and lateral view) of the Müller's percutaneous pinning (a) and Kapandji's Kirschner wire fixation (b).

All procedures were performed under general anesthesia. Single-shot antibiotic prophylaxis was given in each patient. The radius was stabilized with one, two, or three K-wires using the Muller (Fig. 2.) or the Kapandji (Fig. 2.) percutaneous pinning method. In case of a distal forearm fracture a long arm cast was applied of 4 weeks following surgery.

**Figure 3:**
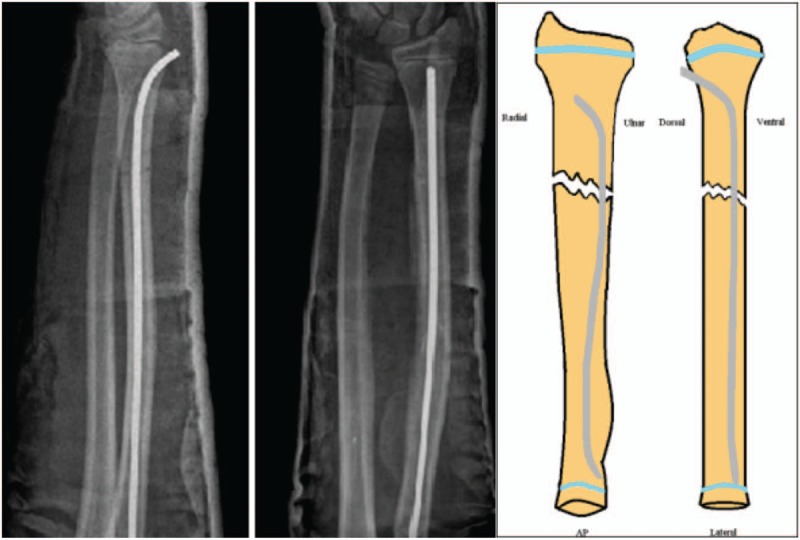
Lateral and anteroposterior radiographs representing the dorsally inserted ESIN (DESIN) technique. DESIN = dorsally inserted titanium elastic stable intramedullary nailing, ESIN = elastic stable intramedullary nailing.

Dorsally inserted radial nails were inserted (approximately 5 mm) over Lister's tubercle, blunt dissection was performed down to bone and an awl was used to make the entry point, without visualization of the extensor tendons. The nails were cut short, just below the level of the skin, to permit hardware removal later on and to avoid injury to the extensor pollicis longus tendon (Fig. 3.). Children treated with DESIN generally did not require cast immobilization.

**Figure 4:**
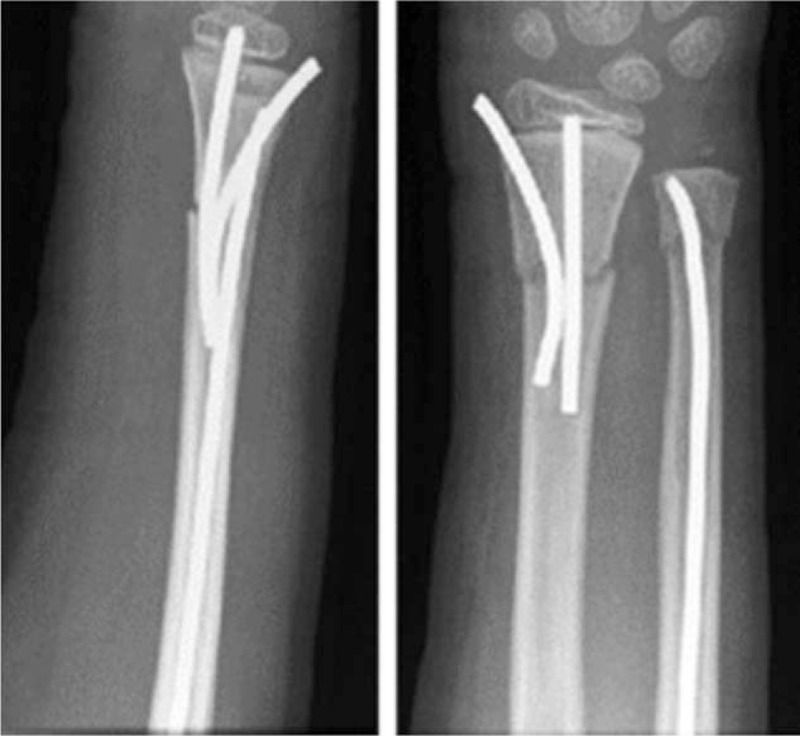
Lateral and anteroposterior radiographs show the SESIN method. SESIN = short titanium elastic stable intramedullary nailing.

During the SESIN technique, the authors used two “C”-shaped pre-bent short titanium elastic nails which were inserted into the distal part of the radius (Fig. 4.) One insertion point was at the distal radial side of the radius, and the other one at the distal dorsal side of the radius just proximal to the growth plate. The second nail was usually thinner and also pre-bent to a “C”-form shape. For the first week a short, removable cast was applied.^[10]^

Gender of the patients, different types of surgical techniques, number of postoperative X-rays, time interval until metal removal, and degree of axial deviation after metal removal were evaluated. The radiological results were assessed for all patients at final follow up. Distance of the fracture line from the radiocarpal surface, the diameter of the distal epiphysis of the radius, and the cumulative diameter of the distal epiphysis of the ulna and radius were measured on the primary X-ray. Based on these parameters, results of the different surgical techniques were analyzed by Kruskal–Wallis test in SPSS program, it is used for comparing two or more independent samples of equal or different sample sizes. We studied the age and gender distribution in the groups.

## 3 Results

We included 196 children. We applied K-wire stabilization in 139 of 196 children (70.0%), DESIN in 44 of 196 children (22.4%), and SESIN in 13 of 196 children (6.6%). With regards to gender distribution, out of the 139 K-wire treated children, 98 were boys and 41 were girls, out of the 44 patients of the DESIN group, 30 were boys and 14 were girls. The ratio was little bit higher in group of SESIN, 10 were boys and 3 were girls. This gender ratio is similar to international and European incidence rates, in which boys are more likely (two times) to be injured. The mean age of the studied children was 10.7^[6–15,25,17]^ years. All groups were the same data and we could be able to compare.

**Figure 5:**
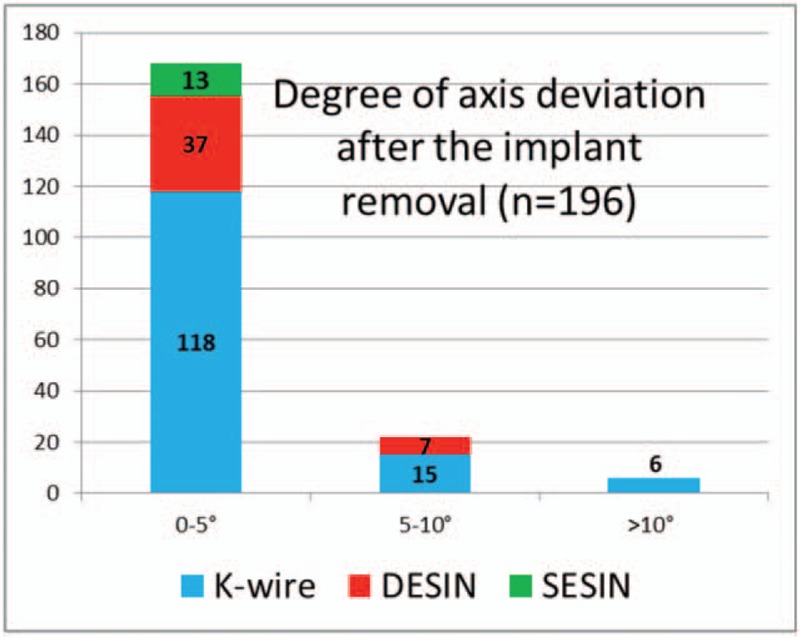
Diagram shows the degree of axis deviation of the radius after implant removal (blue indicates K-wire fixation, red indicates DESIN, and green indicates SESIN). DESIN = dorsally inserted titanium elastic stable intramedullary nailing, K-wire = Kirschner wire, SESIN = short titanium elastic stable intramedullary nailing.

**Figure 6:**
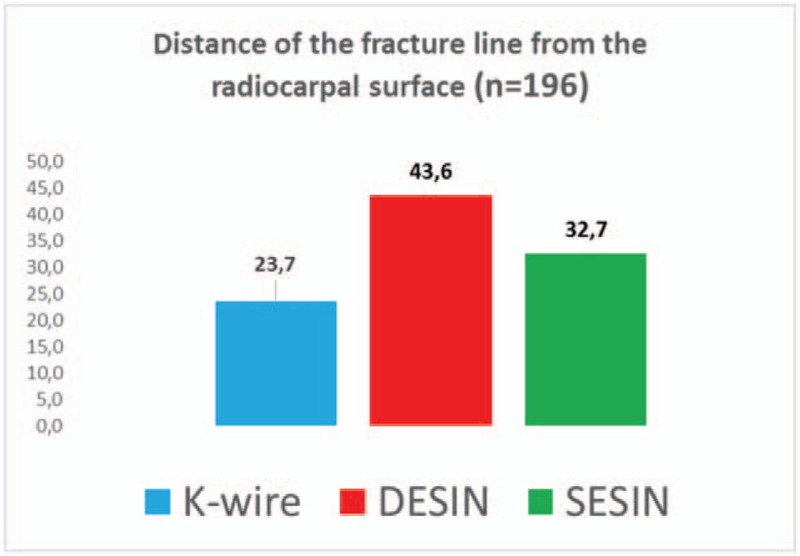
Bar diagram presents the distance (mm) of the fracture line from the radiocarpal surface (blue indicates K-wire fixation, red indicates DESIN, and green indicates SESIN). DESIN = dorsally inserted titanium elastic stable intramedullary nailing, K-wire = Kirschner wire, SESIN = short titanium elastic stable intramedullary nailing.

Casts were used in all patients treated by K-wire fixation. A short removable splint was applied for 1 week in children treated with SESIN. Cast immobilization was not applied for patient treated with SESIN. Control X-ray images were obtained on average in 4.3, 2.6, and in 3.4 times per patient after K-wire, DESIN and SESIN treatment, respectively. The average time until metal removal was significantly shorter (3.8 months), following stabilization with K-wires when compared to the other groups. The time until implant removal was equal in patients treated by different types of elastic nailing, (average 6 months). In children treated with K-wire, axial deviation of <5° was seen in 118 patients, 5° to 10° deviation in 15 patients, while deviation was above 10° in six children. One patient required further reconstruction, due to residual deformity. In the DESIN group, <5° axial deviation was found in 37 patients and 5° to 10° in seven patients. In all 13 children treated with SESIN, axial deviation was measured to be <5° (Fig. 5). The fracture distance from the radiocarpal surface was on average 23.7 mm in the children treated with K-wire, 45.6 mm treated with DESIN and 32.7 mm treated with SESIN, respectively (Fig. 6). Based on Kruskal–Wallis test we found that strong correlation of the chosen fixation method between distal epiphysis of the radius, and the cumulative diameter of the distal epiphysis of the ulna and distance of the fracture line from the radiocarpal surface. In our clinic based on retrospective study results the distance of the fracture line determinate the operative technique.

## 4 Discussion

The current study was designed to evaluate the differences in clinical and radiographic outcomes of dia-metaphyseal fractures of the radius in children between three different (K-wire, DESIN, SESIN) methods of stabilization. Percutaneous pin fixation is most commonly used for unstable distal radial, or distal forearm fractures in pediatric patients. This method has several advantages: it is minimally invasive, has short operative time, implant removal is not technically demanding and it does not require any special instrumentation. K-wire fixation has some complications as well: migration of the wires, damage to the growth plate, skin irritation and infection. It is recommended to combine K-wire fixation with application of a short, or long cast after the reduction and fixation.^[3,15,25]^

Dorsal insertion nailing, DESIN is also an accepted method of fixation for the distal dia-metaphyseal fractures of the radius.^[8,9]^ Generally it is difficult to bend the nail before the nail tip has been advanced into the proximal radius, which is the key point of this method. If the fracture is located too close to the physis the risk of the dorsal cortical damage is relatively high. It is very important to verify the optimal level of the fracture when the DESIN technique is indicated. This type of minimally invasive technique usually does not require cast immobilization.

SESIN technique is a novel method for fixation of the unstable distal dia-metaphyseal radial fracture in children.^[10]^ SESIN procedure has a risk of complications such as: injury of the superficial (sensory) branch of the radial nerve, iatrogenic injury of extensor pollicis longus tendon, and injury of the physis if the insertion points are incorrect. The authors suggested this technique for the dia-metaphyseal fracture because of the fixation in this zone is difficult, usually too distal to treat by DESIN, and too proximal to fix with a K-wire. We conclude that SESIN is an effective, safe and stable form of stabilization for unstable fractures of the distal third of the radius. A number of different treatment options have been published previously in the literature. Some of them suggest the transepiphyseal fixation. The main complication of these procedures is iatrogenic physeal injury and subsequent secondary physeal arrest and deformity.^[20]^ Almost all the previous studies described technical problems and high rates of different complications. Two articles published excellent results after treatment of this type of fracture. One of these technique does not respect the physis.^[25]^ The SESIN technique respects the physis.^[10]^

In this study we focused on the distance of the fracture from the articular surface, which may determine the surgical procedure applied. We were unable to find any previous studies, which focused on the measurement of the fracture distance from the articular surface. Based on our results we found that the fracture distance determines the operative method. If the fracture distance is shorter than the width of the distal epiphysis of the radius the recommended treatment is K-wire fixation based on our retrospective study. If the fracture distance is higher than the cumulative width of the distal epiphysis of the ulna and radius the optimal treatment is DESIN according to our results. In the study period SESIN technique was used just in the distal dia-metaphyseal radial fractures. The limitations of our study are that it was retrospective and conducted in one center. The patients were non-comparative and not randomized. If the level of the fracture distance was measured between the distal radius epiphysis width and cumulative width of the radius and ulna, stabilization was performed with the presented three different methods. K-wire fixation is a gold standard method for the distal metaphyseal radial fracture, some authors suggest treating the transition zone fractures this way as well. Based in our results the SESIN is a safe and physis-sparing procedure for stabilizing the unstable dia-metaphyseal radius fracture. We have not observed any complication for the method in our series. We did not apply SESIN used in any other level fractures, just in the transition zone. Based in our results the fracture distance determined the operative technique. In our department K-wire fixation was indicated, when the fracture distance was shorter than the width of the distal radius. DESIN procedure was chosen when the fracture distance was higher than the cumulative diameter of the radius and ulna. Transition zone fractures were treated differently depending on the surgeon, but the SESIN technique was used predominantly in the fractures of transition zone. There were significant differences between the axial deviations at the time interval until implant removal. The highest incidence of axial deviation was measured in patients treated by K-wiring. The shortest time of the implant removal was measured in K-wire treated group. No further differences were found in our series.

## Author contributions

**Conceptualization:** Gergo Jozsa.

**Formal analysis:** Tamas Juhasz.

**Methodology:** Gergo Jozsa.

**Project administration:** Gergo Jozsa, Gertrud Devecseri.

**Supervision:** Zsolt Juhasz, Peter Vajda, Tamas Juhasz.

**Writing – original draft:** Gergo Jozsa.

**Writing – review & editing:** Gergo Jozsa, Marcell Varga.
